# Molecular characterization and transcriptional regulation analysis of the *Torreya grandis* squalene synthase gene involved in sitosterol biosynthesis and drought response

**DOI:** 10.3389/fpls.2023.1136643

**Published:** 2023-06-20

**Authors:** Feicui Zhang, Congcong Kong, Zhenmin Ma, Wenchao Chen, Yue Li, Heqiang Lou, Jiasheng Wu

**Affiliations:** State Key Laboratory of Subtropical Silviculture, Zhejiang A&F University, Hangzhou, China

**Keywords:** *Torreya grandis*, transcriptional regulation, squalene synthase, sitosterol biosynthesis, drought

## Abstract

The kernel of *Torreya grandis* cv. ‘Merrillii’ (Cephalotaxaceae) is a rare nut with a variety of bioactive compounds and a high economic value. β-sitosterol is not only the most abundant plant sterol but also has various biological effects, such as antimicrobial, anticancer, anti-inflammatory, lipid-lowering, antioxidant, and antidiabetic activities. In this study, a squalene synthase gene from *T. grandis*, *TgSQS*, was identified and functionally characterized. *TgSQS* encodes a deduced protein of 410 amino acids. Prokaryotic expression of the TgSQS protein could catalyze farnesyl diphosphate to produce squalene. Transgenic *Arabidopsis* plants overexpressing *TgSQS* showed a significant increase in the content of both squalene and β-sitosterol; moreover, their drought tolerance was also stronger than that of the wild type. Transcriptome data from *T. grandis* seedlings showed that the expression levels of sterol biosynthesis pathway-related genes, such as *HMGS*, *HMGR*, *MK*, *DXS*, *IPPI*, *FPPS*, *SQS*, and *DWF1*, increased significantly after drought treatment. We also demonstrated that TgWRKY3 directly bound to the *TgSQS* promoter region and regulated its expression through a yeast one-hybrid experiment and a dual luciferase experiment. Together, these findings demonstrate that *TgSQS* has a positive role in β-sitosterol biosynthesis and in protecting against drought stress, emphasizing its importance as a metabolic engineering tool for the simultaneous improvement of β-sitosterol biosynthesis and drought tolerance.

## Introduction

Sterols are isoprenoid-derived molecules found in bacteria, fungi, insects, mammals, and plants (Yuan et al, 2010). In plants, more than 250 kinds of sterols and sterol conjugates have been identified, including free sterols, sterol esters, sterol glucosides, and acylated sterol glucosides. The most abundant plant sterols are sitosterol, followed by stigmasterol and campesterol (Goldstein and Brown, 1990; Schluttenhofer and Yuan, 2015). Sitosterol is not only a component of the plant cell membrane, which plays an important role in seed germination and organ development; it can also participate in the plant’s response to low temperatures, wounds, salt, and drought stress (Elkeilsh et al, 2019). To initiate plant sterol biosynthesis, isopentenyl diphosphate (IPP) and dimethylallyl diphosphate (DMAPP) are generated from either the cytosolic mevalonate (MVA) pathway or the plastidial methylerythritol phosphate (MEP) pathway (Guan et al, 1998; Jiang et al, 2017). Then, the “head-to-tail” condensation of IPP and DMAPP forms C15 farnesyl diphosphate (FPP). Subsequently, two molecules of FPP are condensed head-to-head by squalene synthase (SQS) to form squalene (Nguyen et al, 2013). Finally, sterols, such as sitosterol, stigmasterol, and campesterol, are produced through a series of reactions (Singh et al., 2017). Because FPP is also the precursor of other non-sterol isoprenoids, such as ubiquinones and sesquiterpenoids, regulation of SQS has been considered important for sterol biosynthesis (Nie et al, 2019).

Some studies have shown that *SQS* expression significantly increases in roots, stems, and leaves when *Panax ginseng* and *Portulaca oleracea* are treated with methyl jasmonate (Kumar et al., 2015). In addition, several transcription factors have been reported to regulate *SQS* expression. For example, two bHLH family transcription factors, SREBP1a and SREBP2 (sterol regulatory element binding proteins), are involved in cholesterol synthesis by regulating the expression of *SQS* in human cells (He et al., 2016). TPO1 negatively regulates *Saccharomyces cerevisiae SQS* (*ERG9*) expression, while YER064C and SLK19 positively regulate its expression (Tai et al., 2023; Kennedy and Bard, 2001). WRKY1 increases the content of triterpenes, such as withaferin, by positively regulating *SQS* expression in *Withania somnifera* (Sun et al., 2012).

*Torreya grandis* cv. ‘Merrillii’ is an excellent variety, with a cultivation history of more than one thousand years. The kernel of *T. grandis* cv. ‘Merrillii’ is a precious nut that is rich in unsaturated fatty acids and β-sitosterol, with insecticidal, anti-inflammatory, and antioxidant effects (Huang et al., 2007; Divi and Krishna, 2009). Several studies have indicated that β-sitosterol can protect against breast cancer (Awad et al., 2007; Ju et al., 2004), prostate cancer (Awad et al., 2001), colon cancer (Awad et al., 1997; Clough and Bent, 1998), and gastric cancer (Zhao et al., 2017) by inhibiting cell proliferation and inducing apoptosis. In addition, β-sitosterol can inhibit the increase in blood lipids caused by a high-fat diet (Singh et al., 2015; Salehi et al., 2020). With the given benefits, improving the β-sitosterol content in *T. grandis* can increase the kernel’s nutritional values. In this study, we cloned the *SQS* of *T. grandis* for the first time and proved that it encodes an active squalene synthase. The β-sitosterol content increased significantly in mature seeds of *TgSQS*-overexpressing homozygous lines of *Arabidopsis thaliana*; moreover, their drought tolerance was also stronger than that of the wild type. We also demonstrated that TgWRKY3 directly bound to the *TgSQS* promoter region and regulated its expression through a yeast one-hybrid experiment and a dual luciferase experiment.

## Materials and methods

### Plant materials, growth conditions, and drought treatments

For transcriptome analyses, one-year-old *T. grandis* seedlings were transplanted to pots with 300 g of a soil mixture and grown in a shady plastic greenhouse. After two weeks, the surviving seedlings were irrigated for 12 h, and then irrigation was withdrawn to start drought treatment.

For drought resistance tests, plants were grown in a potting soil mixture in growth chambers at 22°C with 16 h/day illumination. The relative humidity was approximately 70% ( ± 5%). After growing for one week, the plants were irrigated with 1 L of water per tray for 6 h, and then drought treatment was imposed by withdrawing irrigation for half of the plants until most died. The other half were grown under a standard irrigation regime as a control. After 20 days of drought treatment, representative pots were placed to take photos.

For DAB and NBT staining, *Arabidopsis* seeds were surface sterilized and cold treated at 4°C for three days. Then, the seeds were plated on an MS medium containing 3% (w/v) sucrose and 0.8% (w/v) agar and grown at 22°C with a 16-h daily light period. The 10-day-old seedlings were transferred to the MS (Murashige and Skoog) medium with 10% PEG (with controls) for three days before staining. For DAB staining, the samples were immersed in a DAB solution (SL1805, Coolaber) for 4 h and then in 95% ethanol for decoloring. For NBT staining, the samples were first immersed in NBT staining solution (S19048, Yuanye) until a dark blue color appeared (approximately 1 h) and then in 95% ethanol for decoloring. Photographs were taken using a stereo microscope (SZX16, Olympus).

### Phylogenetic analysis

Phylogenetic analysis generated a bootstrap neighbor-joining evolutionary tree using MEGA 7.0 with 1,000 bootstrap replicates. Amino acid sequence alignment was conducted using DNAMAN software.

### Gene cloning, construction, and transformation in A. thaliana

To obtain *TgSQS* overexpression in transgenic plants, the coding sequence of *TgSQS* was PCR-amplified with the primers listed in **Table S1** and introduced into the Super1300 vector driven by a Super promoter (multiple CaMV35S are connected in series). The construct was transformed into *Agrobacterium* strain GV3101 for transfection of *Arabidopsis* using the floral-dip method (Devarenne et al, 2002). Seeds were screened on an MS agar medium containing 50 mg/L of hygromycin. The selected T3-generation transgenic plants with 100% resistance to hygromycin were considered homozygous lines and harvested for further analysis.

### Subcellular localization

The coding sequence of TgSQS was cloned into a modified pCAMBIA1300, which had GFP fused to the N-terminal, and introduced into *Agrobacterium tumefaciens* strain GV3101. The transient gene expression analysis of TgSQS and an endoplasmic reticulum marker in *Nicotiana benthamiana* was performed according to a previous study (Nakashima et al., 1995). Agrobacteria were grown overnight in LB (Luria-Bertani) media and brought to an OD600 of 0.8 in the injection solution. After three days of injection, green fluorescent protein (GFP) and red fluorescent protein (RFP) fluorescence was observed *via* confocal laser scanning microscopy (LSM510, Carl Zeiss).

### Prokaryotic protein expression and enzyme activity analysis

The coding sequence of *TgSQS* was introduced into the pET-32a vector (provided by Zuying Zhang) and transformed into competent Rosetta (DE3) to produce a recombinant protein containing a Trx-His-tag at the N-terminus. The cloning sites were BamHI and SacI. The transformed bacteria were grown to an OD600 of 0.6 at 37°C in an LB medium, and then 0.4 mM isopropyl β-D-1-thiogalactopyranoside (IPTG) was added to induce TgSQS expression. After culturing for 20 h at 16°C, the cells were collected and lysed by sonication. The crude enzyme solution was subjected to Trx-His-Tag purification using a Ni Sepharose 6 Fast Flow gravity column to obtain purified protein. Imidazole was removed from the purified protein using centrifugal ultrafiltration (Amicon Ultra 30 kDa, Millipore, Burlington, MA, USA). After BCA protein quantification, 10-μl samples were used to run a 10% SDS-PAGE gel.

The enzyme reaction of the recombinant protein TgSQS was conducted as follows: 50 µg of purified protein was incubated at 30°C with 20 µg of FPP triammonium salt (Sigma–Aldrich, St. Louis, MO, USA), 50 mM Tris–HCl (pH7.5), 25 mM MgCl_2_, 1 mM DTT, 2% glycine, and 3 mM NADPH in a total reaction volume of 500 µl. After 8 h, the reaction product was extracted three times with 500 µl of n-hexane, concentrated to 100 µl, and analyzed using GC–MS. GC–MS detection was performed using Trace GC Ultra-ISQ (Thermo Scientific) with a DB-5MS column (30 m × 0.25 mm × 0.25 μm; Agilent). The GC temperature program was as follows: 80°C, raised to 300°C at a rate of 15°C/min, and held at this temperature for 18 min. The carrier gas was ultrahigh-purity helium at a flow rate of 1.0 ml/min. The GC interface temperature was 290°C, and the sample injection volume used was 1 μl.

### Squalene analysis

Dry *A. thaliana* seeds were accurately weighed to 0.039 g and ground with liquid nitrogen. Ultrasonically extracted samples were extracted for 20 min three times, and the solvent was removed under low temperature and pressure and dissolved in 1 ml of n-hexane. A total of 1.5 ml of a 2 mol/L KOH-ethanol solution was added to 0.4 ml of the extraction solution. Samples were saponified ultrasonically for 10 min in a 60°C water bath and placed in a 60°C oven for 60 min after vortexing. They were shaken for 1 min, and 1 ml of water and 1 ml of n-hexane were added after cooling. Samples were extracted for 2 min, and then the supernatants were removed. We added 0.25 g of anhydrous sodium sulfate to purify the supernatants, dried the supernatants with liquid nitrogen, and then dissolved the precipitates in 0.1 ml of n-hexane to start GC–MS analysis. The GC–MS detection was performed using a Trace GC Ultra-ISQ (Thermo Scientific) with a DB-5MS column (30 m × 0.25 mm × 0.25 μm: Agilent). The GC temperature program was as follows: 80°C, raised to 300°C at a rate of 15°C/min, and held at this temperature for 18 min. The carrier gas was ultrahigh-purity helium at a flow rate of 1.0 ml/min. The GC interface temperature was 290° C, and the sample injection volume used was 1 μl.

### RNA extraction and transcriptome analysis

Total RNA was isolated from the leaves of *T. grandis* seedlings treated with drought stress using the RNAprep Pure Plant Kit (DP441, Tiangen); mRNA was purified from total RNA using poly-T oligo-attached magnetic beads. First-strand cDNA was synthesized using a random hexamer primer and M-MuLV reverse transcriptase (RNase H-). The library fragments were purified with the AMPure XP system (Beckman Coulter, Beverly, USA) to select cDNA fragments that were preferentially 370–420 bp in length. Library quality was assessed using a Qubit2.0 Fluorometer, Agilent Bioanalyzer 2100 system, and qRT-PCR. The clustering of the index-coded samples was performed on a cBot Cluster Generation System using the TruSeq PE Cluster Kit v3-cBot-HS (Illumia) according to the manufacturer’s instructions. After cluster generation, the library preparations were sequenced on an Illumina Novaseq platform, and 150-bp paired-end reads were generated.

Raw reads of the fastq format were first processed using in-house perl scripts. At the same time, the Q20, Q30, and GC content of the clean data were calculated. Differential expression analysis of two conditions/groups (two biological replicates per condition) was performed using the DESeq2 R package (1.20.0). The obtained P-values were adjusted using the Benjamini–Hochberg method, which is designed to control the false discovery rate. Genes with an adjusted P-value <0.05 found by DESeq2 were assigned as differentially expressed. Gene Ontology (GO) enrichment analysis and KEGG pathways of differentially expressed genes were implemented using the cluster Profiler R package, in which gene length bias was corrected.

### Quantitative real-time PCR (qRT-PCR)

qRT-PCR was performed with a C1000 Touch™ Thermal Cycler system (Bio-Rad) and the ChamQ SYBR qPCR Master Mix kit (Vazyme). The relative expression level was calculated according to the 2^−△△Ct^ method. The actin gene was used as a reference gene. Ct represents the PCR cycle number at which the amount of target reaches a fixed threshold. The corresponding primers are listed in **Table S2.**

### Sterol extraction and analysis

We accurately weighed 0.039 g of dry *A. thaliana* seeds and 0.5 g of fresh leaves (from *T. grandis* seedlings treated with drought stress) and ground them in liquid nitrogen. Samples were extracted with ultrasound for 20 min three times, and the solvent was removed under low temperature and pressure and dissolved in 1 ml of n-hexane. A volume of 1.5 ml of a 2 mol/L KOH-ethanol solution was added to a 0.4 ml extraction solution. Samples were ultrasonically saponified for 10 min in a 60°C water bath and placed in a 60°C oven for 60 min after vortexing. They were shaken for 1 min, and 1 ml of water and 1 ml of n-hexane were added after cooling. Samples were extracted for 2 min, and the supernatants were removed. We added 0.25 g of anhydrous sodium sulfate to purify the supernatants, dried the supernatants with liquid nitrogen, and dissolved the precipitates with 0.1 ml of n-hexane for silylation to form trimethylsilyl ether derivatives of sterols, which were used for GC–MS.

Samples (1 µl) were injected into a GCMS-QP201PLUS apparatus (Shimadzu), which consisted of an automated sampler injection system, a split/splitless injector, and a DB-5MS column (30 m × 0.25 mm × 0.25 μm: Agilent). The GC temperature program was as follows: 80°C, raised to 290°C at a rate of 20°C/min, and held at this temperature for 18 min. The carrier gas was ultrahigh-purity helium at a flow rate of 1.0 ml/min and a 1:10 split injection ratio. Authentic campesterol, stigmasterol, β-sitosterol, and cycloartenol were purchased from Sigma-Aldrich. For quantification, a standard curve for individual authentic standards was generated.

### Vector construction and dual luciferase assay

The coding sequence of *TgWRKYs* was obtained from RNA sequencing and cloned into the pCAMBIA 1300-GFP vector to express the effector. The 1870-bp promoter fragments of *TgSQS* were sub-cloned into the pGreenII 0800-LUC vector as a reporter. The corresponding primers are listed in **Table S1**. Individual effector vectors and recombinant reporter vectors were transferred to *A. tumefaciens* GV3101. The reporter was mixed with each kind of effector or the empty pCAMBIA 1300-GFP vector (control) at a 1:1 (v:v) ratio and then injected into tobacco leaves as described previously (Nakashima et al., 1995). A dual-luciferase assay was carried out using the Dual-Lumi™ Luciferase Reporter Gene Assay Kit (Beyotime, RG089). The firefly luciferase (FLUC) and Renilla luciferase (RLUC) values were measured using a Promega GloMax20/20 Luminescence Detector. The relative LUC activity was calculated as the ratio between FLUC and RLUC activities, and three biological replicates were applied.

### Yeast one-hybrid assay

Yeast one-hybrid (Y1H) assays were carried out with the Matchmaker Gold Yeast One-Hybrid System Kit (Clontech) according to the manufacturer’s protocol (PT4087-1, Clontech, USA). The corresponding primers are listed in **Table S1**. Based on the distribution of predicted W-box binding sites in the *TgSQS* promoter region, three short fragments of the *TgSQS* promoter (the adenine residue of the translational start codon ATG) were assigned position +1, and the numbers flanking the sequences of the *TgSQS* promoter fragments were counted based on this number. The positions of three 200-bp sequences containing a W-box were as follows: W1, 1775 to 1575 bp; W2, 1275 to 1075 bp; and W3, 375–175 bp). They were subcloned into the pAbAi vector to obtain *pAbAi-W1/W2/W3*. **Figure S2** provides the sequence of the *TgSQS* promoter. The coding sequence of *TgWRKY3/6/7* was inserted into the pGADT7 vector to construct the prey-AD vector. Then, the linearized *pAbAi-W1/W2/W3* vector was transformed into Y1HGold. After determining the minimal inhibitory concentration of Aureobasidin A (AbA) for positive transformants, the prey-AD vector was transformed into the bait yeast strain. Successfully transformed yeast strains were grown on the corresponding SD medium (SD/−Leu, SD/−Leu + AbA) for 3–5 days to take photos.

## Results

### Identification of TgSQS

**Figure 1:**
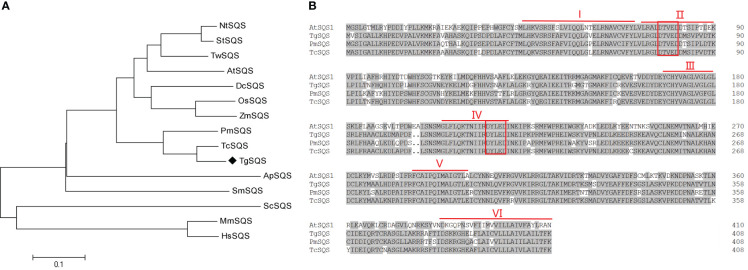
Analysis of the TgSQS amino acid sequence. **(A)** Construction of the phylogenetic tree of squalene synthase from different species using the neighbor-joining method. The accession numbers of the genes used were: NtSQS, *Nicotiana tabacum*, AAB08578.1; StSQS, *Solanum tuberosum*, BAA82093.1; AtSQS1, *Arabidopsis thaliana*, AEE86403.1; OsSQS, *Oryzasativa Japonica* Group, BAA22557.1; DcSQS, *Dendrobium catenatum*, AGI56082.1; ZmSQS, *Zea mays*, BAA22558.1; PmSQS, *Pinus massoniana*, AHI96421.1; TcSQS, *Taxus cuspidate*, ABI14439.1; ApSQS, *Auxenochlorella protothecoides*, KFM22694.1; HsSQS, *Homo sapiens*, AAB33404.1; ScSQS, *Saccharomyces cerevisiae*, AAA34597.1; TwSQS, *Tripterygium wilfordii*, AMR60779.1; SmSQS, *Selaginella moellendorffii*, XP024532546.1; MmSQS, *Mus musculus*, AP_034321.2. **(B)** Alignment of the full-length amino acid sequences of TgSQS and its homologs in *Arabidopsis thaliana*, *Taxus cuspidate*, and *Pinus massoniana*. The six conserved regions (I, II, III, IV, V, and VI) of squalene synthase are marked by red lines, and two aspartate rich domains (DXXXD) are marked by red squares.

Since the genome of *T. grandis* has not yet been sequenced, we searched the transcriptome data (NCBI accession Nos. SRX9417001–SRX9417009) and found a unigene annotated as squalene synthase. This sequence was used as a query in the blastx of NCBI, and the results showed that this unigene had high identity (>80%) with squalene synthase from *Taxus cuspidate*, *Ginkgo biloba* L., and *Pinus massoniana*, indicating that this unigene likely encodes squalene synthase in *T. grandis*; thus, it was named *TgSQS*. The 1230-bp coding length of *TgSQS* was determined using sequence alignment in blastx. Phylogenetic tree analysis of TgSQS with SQSs from angiosperms, gymnosperms, algae, and animals showed that TgSQS was most closely related to gymnosperms, such as *T. cuspidate*, and furthest from animals (**Figure 1A**). Multiple alignments of TgSQS with squalene synthase from *A. thaliana*, *T. cuspidate*, and *P. massoniana* showed that there were six highly conserved regions, I–VI, in its amino acid sequence, among which II and IV contained aspartic acid (DXXXD) active sites (**Figure 1B**), which were reported to bind to isopentene phosphate groups (Rushton et al., 2010), and VI was reported to perform the function of membrane targeting and anchoring (Ishihama et al., 2011). The DeepTMHMM-based predictions of transmembrane domains revealed that TgSQS had one C-terminal transmembrane domain (amino acid residues 389–407), which was highly like those predicted for *A. thaliana* (**Figure S1B, C**) and *Taraxacum koksaghyz* SQS (Valitova et al, 2016). We further investigated the subcellular localization of TgSQS through transient expression in *N. benthamiana* leaves. As can be seen in **Figure S1A**, the TgSQS protein fused with GFP at the N-terminal colocalized with the endoplasmic reticulum marker, which was consistent with the literature (Valitova et al., 2016). These results suggest that *TgSQS* encodes functional squalene synthase.

### Functional characterization of TgSQS *in vitro* and *in vivo*

**Figure 2:**
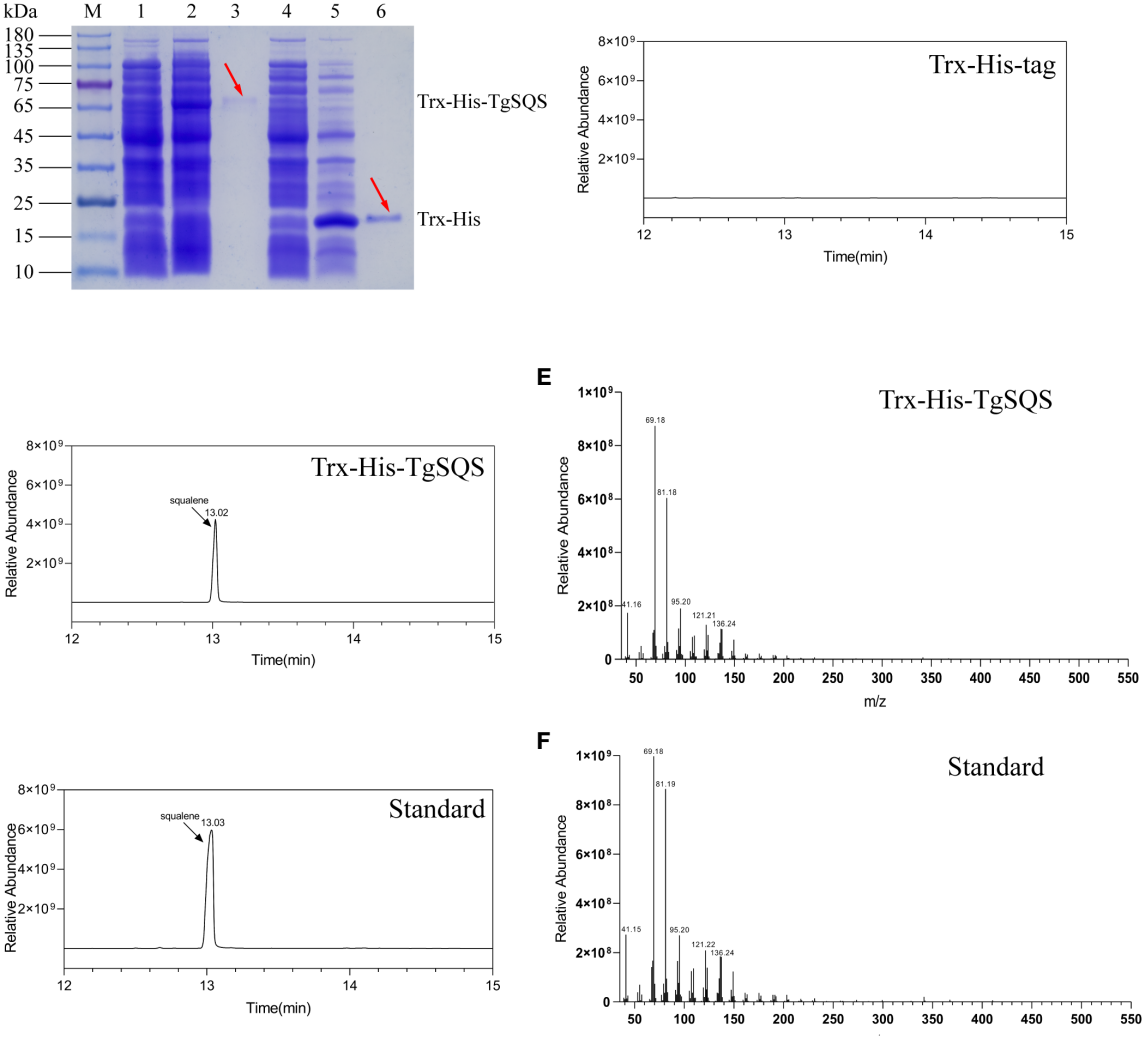
Identification of recombinant TgSQS protein and gas chromatography-mass spectroscopy (GC–MS) analysis of squalene in the catalytic products of TgSQS. **(A)** 10% SDS-PAGE detection of recombinant TgSQS protein expressed in **(*E*)** *coli* Rosetta (DE3). M, protein marker; Line 1, induced *pET-32a-TgSQS* bacteria; Line 2, supernatant of induced *pET-32a-TgSQS* bacteria after ultrasound; Line 3, purified *pET-32a-TgSQS* protein; Line 4, induced *pET-32a* bacteria; Line 5, supernatant of induced *pET-32a* bacteria after ultrasound; Line 6, purified Trx-His-Tag; **(B)** GC–MS detection of the reaction products of Trx-His-TgSQS; **(C)** GC–MS detection of the standard squalene; **(D)** GC–MS detection of the reaction products of Trx-His-Tag (control); **(E)** MS analysis of the catalytic products of Trx-His-TgSQS; **(F)** MS analysis of standard squalene.

To understand the catalytic function of TgSQS, the recombinant expression plasmid *pET-32a-TgSQS* was constructed and transformed into *E*. *coli* Rosetta (DE3) to produce a recombinant protein containing a Trx-His-tag at the N-terminus. The *pET-32a* empty vector served as a control. After induced expression, extraction, and purification, the purified recombinant proteins were analyzed using SDS-PAGE. TgSQS was successfully extracted from the supernatant of lysates with a molecular weight of around 70 kDa (**Figure 2A**). The enzyme activity of TgSQS was assayed using the purified protein with the cofactors NADPH and Mg^2+^, while Trx-His-tag was used as a control. The catalytic products were analyzed using GC–MS; the peak at 13.02 min in the profile of TgSQS reaction products (**Figure 2B**) corresponded to that observed in the standard squalene sample (**Figure 2C**), but no such peak was detected in the control (**Figure 2D**). Moreover, the characteristics of peaks in the mass spectrum of TgSQS (**Figure 2E**) were consistent with those of standard squalene (**Figure 2F**). These results proved that TgSQS has the catalytic activity of squalene synthase *in vitro*.

**Figure 3:**
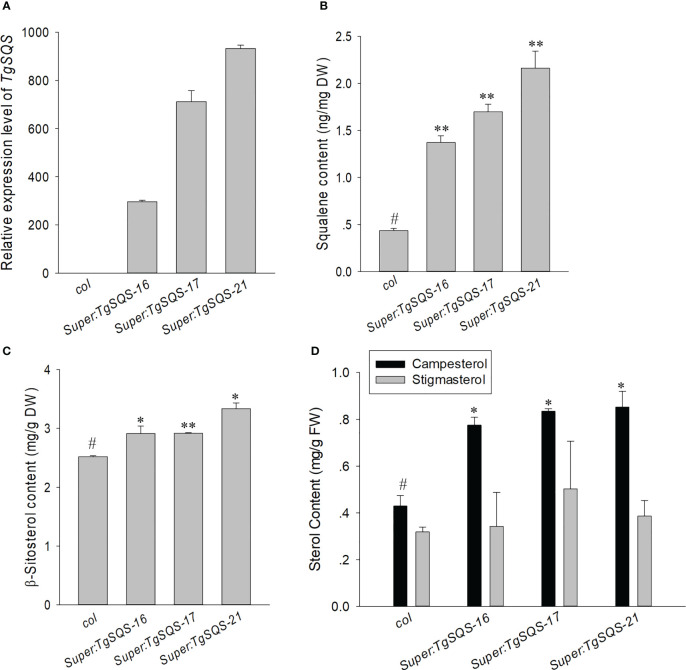
Increased squalene and β-sitosterol content in the seeds of *TgSQS*-overexpressing *Arabidopsis thaliana* lines. **(A)** Transcript level of *TgSQS*, **(B)** seed squalene content, **(C)** seed β-sitosterol content, and **(D)** stigmasterol and campesterol content in *col* and three *TgSQS*-overexpressing *Arabidopsis thaliana* lines. Data are mean ± SD (n = 3); asterisks indicate significant differences relative to the control by a two-tailed Student’s *t*-test. #, control*p <0.05; **p <0.001.

To further verify the function of *TgSQS*, the overexpression plasmid *super1300-TgSQS* was transferred into *A. thaliana*, and three constitutive overexpression lines with significantly higher expression levels of *TgSQS* were obtained (**Figure 3A**). The content of squalene in mature seeds was detected, and the results showed that the content of squalene in the three overexpression lines was significantly higher than that of the wild type (**Figure 3B**), indicating that TgSQS also has the catalytic activity of squalene synthase *in vivo*.

### Overexpression of TgSQS increased β-sitosterol content in Arabidopsis seeds

In plants, the dominant sterols comprise β-sitosterol, campesterol, and stigmasterol, while β-sitosterol is the most abundant. It has been evidenced in many *in vitro* and *in vivo* studies that β-sitosterol possesses various biological actions, such as immunomodulatory, antimicrobial, anticancer, anti-inflammatory, lipid-lowering, antioxidant, and anti-diabetic activities (Babu and Jayaraman, 2020). Therefore, increasing the β-sitosterol content in *T. grandis* seeds is very important to improve their nutritional value. As it is difficult to achieve genetic transformation in *T. grandis*, to verify the role of *TgSQS* in the sterol biosynthetic pathway, we used the super promoter to express *TgSQS* in *A. thaliana* (**Figure 3A**) and tested the β-sitosterol content in mature seeds, as can be seen from **Figure 3C**, which indicates that the β-sitosterol content in the mature seeds of three *TgSQS*-overexpressing lines increased by 15%, 15%, and 30% compared with the wild type, indicating that constitutively increasing *TgSQS* expression in *Arabidopsis* significantly increased β-sitosterol content in mature seeds. At the same time, the campesterol and stigmasterol content were also detected in these materials (**Figure 3D**); the results showed that the campesterol content in three *TgSQS*-overexpressing lines were also significantly higher than that of the wild type. However, the stigmasterol content showed no difference between the three *TgSQS*-overexpressing lines and the wild type.

### TgWRKY3 bound the W-box elements in the TgSQS promoter

**Figure 4:**
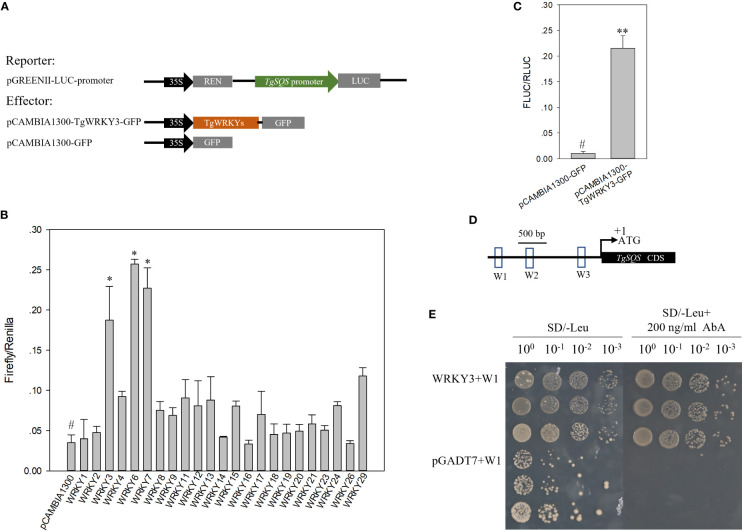
TgWRKY3 directly bound to the promoter of *TgSQS* and positively regulated *TgSQS* expression in tobacco leaves. **(A)** Schematic diagrams of vectors used for the dual luciferase assay; **(B)** Effects of TgWRKYs on the activities of the *TgSQS* promoter. Each column represents the mean ± SD of three biological replicates. Asterisks indicate significant differences relative to the control as determined by a two-tailed Student’s *t*-test. #, control; *p <0.05. **(C)** Effects of TgWRKY3 on the activities of the *TgSQS* promoter. Data are the mean ± SD (n = 4); asterisks indicate significant differences relative to the control by a two-tailed Student’s *t*-test. **p < 0.001. **(D)** Cis-element analysis in the sequence of the *TgSQS* promoter (the bait); W1, W2, and W3 represent 200-bp nucleotide sequences containing the W-box. The nucleotide sequences of W1, W2, and W3 are shown in **Figure S2**. **(E)** Y1H verification of the interaction between TgWRKY3 and the *TgSQS* promoter. The pAbAi vector carrying W1, W2, and W3 (200-bp sequences containing the W-box from the *TgSQS* promoter) and the pGADT7-TgWRKY3 recombinant vector were used to co-transfect Y1HGold receptive cells. Y1HGold cells co-transfected with pAbAi-W2/W3 and pGADT7-TgWRKY3 did not grow normally on the SD/−Leu medium supplied with 200 ng/ml AbA; thus, the pictures were not shown. From left to right, the dilutions of the bacterial solution are 1, 0.1, 0.01, and 0.001. Three monoclonal yeast strains are repeated.

Due to the important role of *TgSQS* in sterol biosynthesis, we studied its transcriptional regulation mechanism. Through cloning and analysis of its promoter (1,860 bp before ATG), three W-box cis-acting elements were found in the entire promoter region. Since the W-box element was the DNA binding site of WRKY family transcription factors, WRKY TFs were regarded as candidates to regulate *TgSQS* expression. In total, 23 *TgWRKYs* with full-length coding sequences were found by searching the transcriptome data of *T. grandis* (NCBI accession Nos. SRX9417001–SRX9417009). All of them were constructed in the overexpression vector *pCAMBIA1300-GFP*, and the dual luciferase assay was carried out with the empty vector as the control. TgWRKY3, TgWRKY6, and TgWRKY7 significantly increased the expression level of the reporter gene (**Figures 4B, C**), indicating that they may regulate the expression of *TgSQS*.

To verify whether TgWRKY3, TgWRKY6, and TgWRKY7 could directly bind to the promoter of *TgSQS*, a yeast one-hybrid experiment was carried out. In the Y1H assay, the pAbAi vector carrying W1, W2, and W3 (200-bp sequences containing W-box from the *TgSQS* promoter, **Figures 4D**, **S2**) and the pGADT7-TgWRKY3/6/7 recombinant vector served as the reporter and the effector, respectively. Only Y1HGold yeast co-transformed with pGADT7-TgWRKY3 and pAbAi-W1 grew normally on the SD/−Leu medium supplied with 200 ng/ml AbA, whereas the yeast carrying the pGADT7-TgWRKY6/7 and pGADT7 empty vector did not (**Figure 4E**), indicating that TgWRKY3 directly bound the W-box-containing region in the *TgSQS* promoter.

### Sterol biosynthesis-related genes in *T. grandis* can respond to drought stress

**Figure 5:**
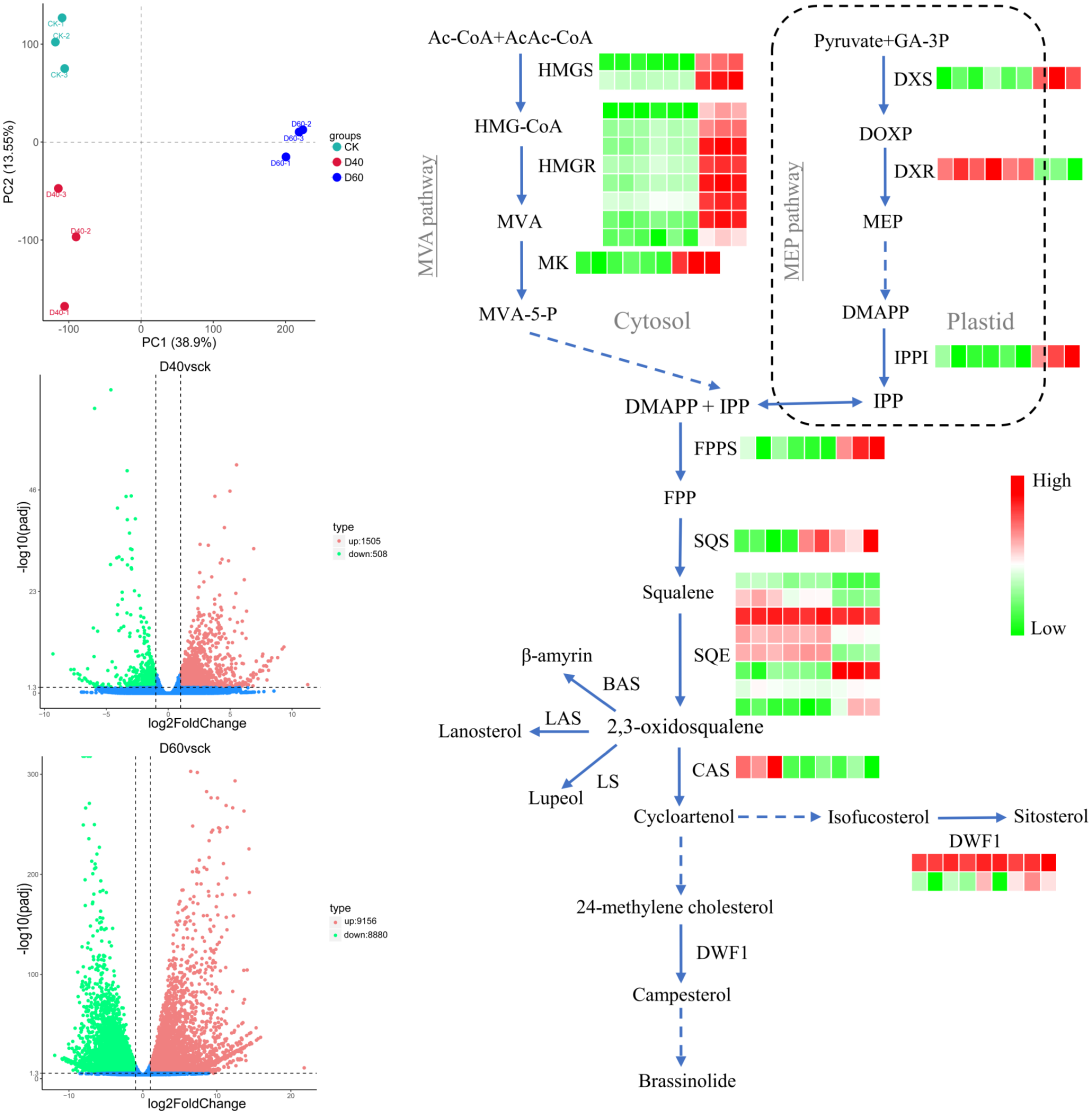
Sterol biosynthesis-related genes in *Torreya grandis* responded to drought stress. **(A)** Principal component analysis (PCA) of the genes. **(B)** Volcano plots for DEGs from CKvsD40; **(C)** Volcano plots for DEGs from CKvsD60. **(D)** Simplified view of isoprenoid biosynthesis in plants. Solid blue arrows indicate single-step reactions, dashed arrows denote several steps, and the double arrow between the cytosolic and plastid compartments indicates metabolic crosstalk between them. In the heat map, the first three squares from left to right represent CK samples, the three in the middle represent D40 samples, and the three on the right side represent the three D60 samples. Ac-CoA, acetyl CoA; AcAc-CoA, acetoacetyl CoA; BAS, b-amyrin synthase; BR6OX2, brassinosteroid-6-oxidase 2; CAS, cycloartenol synthase; DMAPP, dimethylallyl diphosphate; DWF1, D24 sterol reductase; DOXP, 1-deoxy-D-xylulose 5-phosphate; DXR, 1-deoxy-Dxylulose 5-phosphate reductoisomerase; DXS, 1-deoxy-D-xylulose-5-phosphate synthase; FPP, farnesyl diphosphate; FPPS, FPP synthase; GA-3P, glyceraldehyde 3-phosphate; HMG-CoA, 3-hydroxy-3-methylglutaryl-CoA; HMGR, HMG-CoA reductase; HMGS, HMG-CoA synthase; IPP, isopentenyl diphosphate; IPPI, isopentenyl diphosphate isomerase; LAS, lanosterol synthase; LS, lupeol synthase; MEP, 2-C-methyl-D-erythritol 4-phospahte; MVA, mevalonate; MVA-5-P, 5-phosphomevalonate; MK, mevalonate kinase; SQE, squalene monooxygenase/epoxidase; SQS, squalene synthase.

Preliminary experimental results showed that *T. grandis* seedlings had strong drought tolerance. Therefore, we carried out drought treatment on *T. grandis* seedlings and sampled the leaves at the D40 stage (drought treatment for 40 days when soil moisture was zero but seedlings stayed green) and D60 stage (drought treatment for 60 days when seedlings became wilted) to perform transcriptome sequencing with normally watered seedlings (CK, sampled at the D60 stage) as a control. PCA showed low variability among biological repeats, which indicated that there was a high correlation between repetitions (**Figure 5A**). The nine libraries produced over 6G of clean bases, with Q30 percentages (percentage of sequences with sequencing error rates <0.1%) ranging from 94% to 95% (**Table S3**). All bases were assembled into 66,798 unigenes with a mean length of 1,241 bp (**Table S4**). The GO, NT, NR, SwissProt, PFAM, KO, and KOG databases were used to annotate the predicted protein sequence, and 33,318 unigenes were annotated by at least one database (**Table S5**). These data showed that RNA-seq was of high quality and could be used for further analysis.

**Figure 6:**
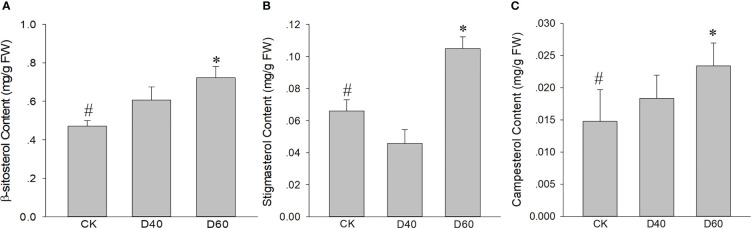
Increased **(A)** β-sitosterol content, **(B)** stigmasterol content, and **(C)** campesterol content in drought-treated *Torreya grandis* seedlings. Data are the mean ± SD (n = 3); asterisks indicate significant differences relative to the control by a two-tailed Student’s *t*-test. #, control; *p <0.05.

A total of 2,013 DEGs, including 1,505 up and 508 downregulated genes, were identified in the D40 stage compared with CK (**Figure 5B**). In the D60 stage, compared with CK, 9,156 genes were upregulated and 8,880 genes were downregulated (**Figure 5C**), indicating that there were many more DEGs in the latter. Moreover, the DEGs of D40vsCK and D60vsCK did not completely overlap. As shown in **Figure S6**, a total of 1,336 DEGs overlapped, of which 461 were jointly upregulated and 266 were jointly downregulated. Brassinosteroid biosynthesis and terpenoid backbone biosynthesis were found in the KEGG path enrichment scatter plot at the D60 stage compared with CK (**Figures S4, S5**), indicating that genes related to terpenoid and sterol biosynthesis were involved in the response of *T. grandis* to drought stress, considering that campesterol is a precursor of brassinosteroids. Consequently, we combined the sterol biosynthesis pathway with the *T. grandis* transcriptome data in this study to draw a simplified map and mark the expression changes of related genes in CK, D40, and D60 stages in the form of a heat map (**Figure 5D**). The expression levels of *HMGS*, *HMGR*, *MK*, *DXS*, *IPPI*, *FPPS*, *SQS*, and *DWF1* increased significantly at the D60 stage (**Table S6**). At the same time, the sterol content in the leaves of the CK, D40, and D60 stages was detected, and the results showed that the content of sitosterol, campesterol, and stigmasterol in the D60 stage was significantly higher than in the CK, while there was no significant difference between the D40 stage and the CK (**Figure 6**). This result was consistent with the fact that the differential genes of the D40 stage in the transcriptome were far less than those in the D60 stage, which might be because the soil moisture at the D40 stage had just dropped to zero and the *T. grandis* seedlings were still in the early stages of drought stress. Together, these results indicate that the sterol biosynthesis pathway is involved in the response of *T. grandis* to drought stress.

### TgSQS responds to drought stress by affecting ROS accumulation in A. thaliana

**Figure 7:**
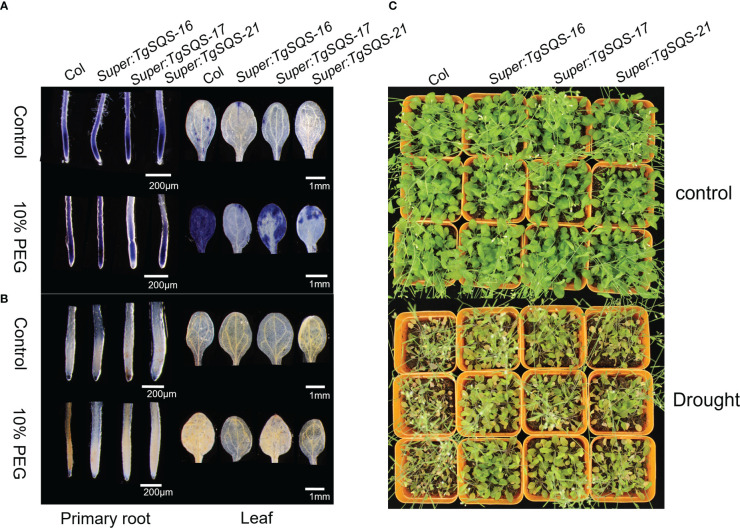
*TgSQS* responded to drought stress by affecting ROS accumulation in *Arabidopsis thaliana*. **(A)** Nitroblue tetrazolium (NBT) staining in the primary root tip and leaf of *TgSQS-OE* and *col* 13-day-old seedlings after treatment with 10% PEG for three days. The color strength shows the O^2−^ concentration in the root tips or leaves. **(B)** 3,3-Diaminobenzidine (DAB) staining in the primary root tip and leaf of *TgSQS-OE* and *col* 13-day-old seedlings after treatment with 10% PEG for three days. The color strength shows the H_2_O_2_ concentration in the root tips or leaves. **(C)** The phenotypes of *TgSQS-OE* and *Arabidopsis col* seedlings under drought stress treatment. Three-week-old *col* and *TgSQS-OE* seedlings were subjected to drought stress without water for 20 days.

To verify the function of *TgSQS* under drought stress, three homozygous overexpressing *A. thaliana* lines and the wild type were used for drought treatment. After 20 days of drought, the wild-type leaves began to wilt, while the leaves of *TgSQS*-overexpressing lines stretched normally, indicating that they were more drought-tolerant than the wild type (**Figure 7C**). To deeply understand the drought tolerance mechanism, the accumulation of H_2_O_2_ and superoxide in the overexpressing lines and the wild type after simulated drought stress was observed by DAB and NBT staining. In this study, 10-day-old seedlings were treated with 10% PEG for 72 h, followed by 3,3-diaminobenzidine (DAB) staining to determine the presence of H_2_O_2_ and nitro blue tetrazolium (NBT) staining to show the presence of the superoxide anion. As shown in **Figures 7A, B**, under control conditions, wild-type and transgenic plants showed similar basal levels of H_2_O_2_ and superoxide, but under 10% PEG treatment, TgSQS-OE leaves and roots showed less H_2_O_2_ and superoxide accumulation than wild-type leaves and roots. These results suggest that *TgSQS* improves drought tolerance by reducing ROS accumulation.

## Discussion

In this study, we found that a unigene in the transcriptome data of *T. grandis* might be the gene encoding functional squalene synthase, and we verified its function *in vitro* and *in vivo* (**Figures 1** **–** **3**). Since *AtSQS* was cloned in 1995 (Nguyen et al., 2013), the *SQS* of many species, such as *Tripterygium wilfordii* (Zhang et al., 2016), soybean (Nguyen et al., 2013), *P. ginseng* (Kim et al., 2011), *Solanum nigrum* (Susan et al., 1991), *Siraitia grosvenorii* (Zhou et al., 2012), birch (Zhao et al., 2009), and persimmon (Zhou et al., 2012), have been cloned. Based on these reports, the number of *SQS* varies among different species, which may be a way for plants to meet changes in different developmental and environmental situations. Several literature reports have shown that SQS is a key enzyme for sitosterol synthesis, and changes in both SQS activity and gene expression levels can affect the phytosterol content. For example, treatment of tobacco suspension cells with SQS inhibitors and fungal elicitors could reduce their SQS activity, thereby significantly reducing their sterol content (Ding et al., 2020; Wollam and Antebi, 2011). The content of β-sitosterol significantly increased after *PgSQS* overexpression in *P. ginseng* and *Acanthopanax senticosus* (Li et al., 2019; Sikder et al., 2014). After reducing the expression of *SQS* by virus-induced gene silencing in *W. somnifera* leaves, a significant decrease in sterol content was detected (Singh et al., 2017). In this paper, we demonstrated that *TgSQS* overexpression in *Arabidopsis* significantly increased the content of β-sitosterol and campesterol in mature seeds (**Figure 3**), which was consistent with the literature (Nguyen et al., 2013), suggesting that we could increase the content of β-sitosterol and campesterol in *T. grandis* nuts to improve its nutritional value by increasing *TgSQS* expression.

Until now, only WsWRKY1 had been known to regulate *WsSQS* and *WsSQE* in *W. somnifera*, according to reports on transcriptional regulation of *SQS* (Sun et al., 2012). In the present study, we demonstrated that TgWRKY3 directly bound to the promoter region of *TgSQS* and upregulated its expression (**Figure 4**). WRKY domain-containing genes comprise one of the largest TF families in plants and are characterized by a highly conserved WRKYGQK motif at the N-terminal end, together with a novel zinc-finger-like motif (Ferrer et al., 2017). In *A. thaliana*, the WRKY family contains 72 members, which can be divided into three categories, and the second category can be further divided into five subclasses (a–e) (Rushtone et al., 2010). The phylogenetic tree analysis of TgWRKY3, WsWRKY1, and WRKY TFs in *A. thaliana* showed that TgWRKY3 has a close relationship with AtWRKY21, AtWRKY74, and AtWRKY39 in Group II-d (**Figure S3**) and that WsWRKY1 has a close relationship with AtWRKY41 and AtWRKY53 in Group III, indicating that we found another WRKY that regulates *SQS* expression. Multiple sequence alignment results showed that TgWRKY3 has a WRKYGQK conserved domain and the characteristic zinc finger domain C2H2, indicating that *TgWRKY3* indeed belongs to the WRKY TFs.

**Figure 8:**
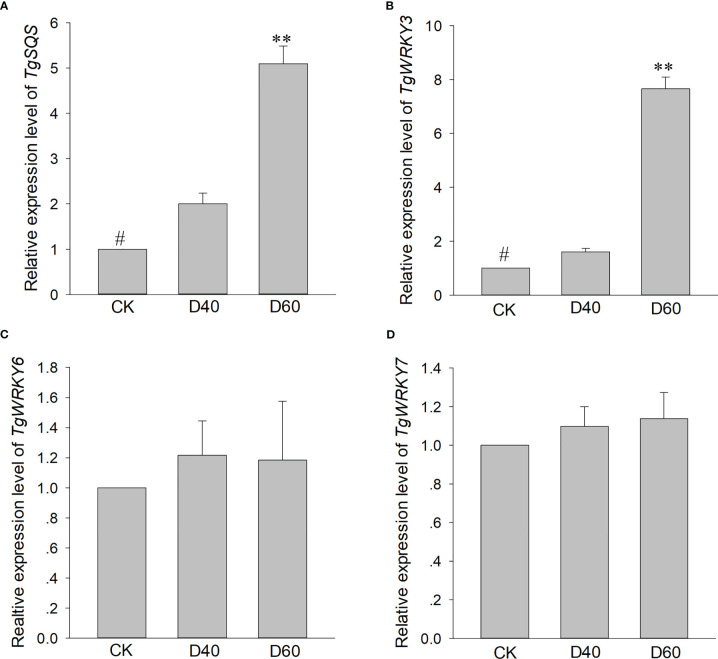
qRT-PCR analysis of gene expression patterns of *TgSQS* **(A)**, *TgWRKY3* **(B)**, *TgWRKY6* **(C)**, and *TgWRKY7* **(D)** among drought treatment stages. Data are the mean ± SD (n = 3); asterisks indicate significant differences relative to the control by a two-tailed Student’s *t*-test. #, control; **p <0.0001.

As one of the largest transcription factor families in plants, WRKY TFs have been found to play a role in plant development and tolerance to a variety of abiotic stressors, including wounding, drought, salt, heat, and cold pressure (Choi et al., 2003; Ju et al., 2004; Wentzinger et al., 2002; Chen et al., 2012). Recent research has also shown that WRKY TFs can participate in regulating the biosynthesis of multiple metabolites, such as sesquiterpenes, alkaloids, and terpenes (Seo et al, 2005). For example, MrWRKY1 interacts with the promoter of *MrFPS* to regulate the biosynthesis of α-Bisabolol in *Matricaria recutita L*. (Unland et al., 2018), and NbWRKY8 can regulate capsidiol biosynthesis by binding the promoter of NbHMGR2 in *N. benthamiana* (Jadaun et al., 2017). In our study, TgWRKY3, TgWRKY6, and TgWRKY7 both significantly increased the expression level of the reporter gene, but only TgWRKY3 directly bound to the promoter of TgSQS (**Figure 4**), indicating that TgWRKY6 and TgWRKY7 regulate *TgSQS* in a manner that interacts with other transcription factors. However, the expression level of *TgWRKY3* in *T. torreya* seedlings after drought treatment significantly increased, while *TgWRKY6* and *TgWRKY7* did not (**Figures 8B–D**), suggesting that TgWRKY3 is involved in both the synthesis of β-sitosterol and the plant’s response to drought stress, while TgWRKY6 and TgWRKY7 might also regulate *TgSQS* and be involved in the response to different stressors.

In this study, we found that *TgSQS*-overexpressing lines showed stronger drought tolerance and less ROS accumulation than the wild type (**Figure 7**), indicating that *TgSQS* probably responded to drought stress by reducing ROS accumulation. However, the specific mechanism is still unclear. Our results also showed that the content of phytosterols (β-sitosterol, campesterol, and stigmasterol) in *T. grandis* seedlings after drought treatment was significantly higher than that in the control; furthermore, the β-sitosterol content was at least one order of magnitude higher than that of campesterol and stigmasterol (**Figure 6**). Studies have shown that an increase in β-sitosterol content can be observed in rice under drought conditions (Lee et al., 2004; Kumar et al., 2015). Additionally, drought tolerance and total antioxidant capacity were significantly improved with 100 µM sitosterol treatment in *T*. *aestivum* and 120 µM sitosterol treatment in *Trifolium repens* (Eulgem et al., 2000; Lou et al., 2019). These results imply that β-sitosterol is involved in the response to drought stress. β-sitosterol has been reported as a component of the cell membrane (Vriet et al., 2012); thus, the response of β-sitosterol to drought is probably realized by changing the membrane fluidity and permeability. However, the effect of *TgSQS* on ROS accumulation is perhaps related to brassinosteroids (BRs). As a plant hormone, BRs control several traits of agronomic importance, such as seed germination, plant architecture, seed yield, and tolerance to various abiotic and biotic stressors (Du et al, 2022; Wani et al., 2021). Several studies have shown that plants treated with exogenous BRs are drought tolerant, and in tomato, it has been clearly demonstrated that a rise in the level of BR biosynthesis is the key to enhancing the tolerance capacity (Nolan et al., 2020; Pandit et al., 2000). In another study, BR treatment rescued the heavy aggregation of ROS in the case of drought stress (Zhang et al., 2018). Therefore, *TgSQS* might affect ROS accumulation by regulating BR biosynthesis.

## Data availability statement

The datasets presented in this study can be found in online repositories. The names of the repository/repositories and accession number(s) can be found below: https://www.ncbi.nlm.nih.gov/ , SAMN32775921 https://www.ncbi.nlm.nih.gov/, SAMN32775922 https://www.ncbi.nlm.nih.gov/, SAMN32775923 https://www.ncbi.nlm.nih.gov/, SAMN32775924 https://www.ncbi.nlm.nih.gov/, SAMN32775925 https://www.ncbi.nlm.nih.gov/, SAMN32775926 https://www.ncbi.nlm.nih.gov/, SAMN32775927 https://www.ncbi.nlm.nih.gov/, SAMN32775928 https://www.ncbi.nlm.nih.gov/, SAMN32775929.

## Author contributions

FZ conceived and guided the experiments, analyzed the data, and wrote the draft. CK performed the experiment and analyzed the data. ZM performed the experiment. WC performed the experiment. YL performed the experiment. HL conceived and guided the experiments. JW is responsible for resources and supervision. All authors contributed to the article and approved the submitted version.
