# Supplementary material for: Molecular characterization and transcriptional regulation analysis of the Torreya grandis squalene synthase gene involved in sitosterol biosynthesis and drought response
Source: Front Plant Sci. 2023 Jun 20;14:1136643. doi: 10.3389/fpls.2023.1136643 (PMC10318344; doi:10.3389/fpls.2023.1136643)
Supplement: Supplementary file 1 [file DataSheet_1.docx]

# Molecular characterization and transcriptional regulation analysis of *Torreya grandis* squalene synthase gene involved in sitosterol biosynthesis and drought response

Feicui Zhang^b #^, Congcong Kong^a #^, Zhenmin Ma^a^, Wenchao Chen^a^, Yue Li^a^, Heqiang Lou^a*^, Jiasheng Wu^a*^

^#^ These two authors contributed equally to this work.

*** Correspondence:**

E-mail addresses: [wujs@zafu.edu.cn](mailto:wujs@zafu.edu.cn) (J. Wu), [20170030@zafu.edu.cn](mailto:20170030@zafu.edu.cn) (H. Lou)

Table S1. Primers of sequences for vector construction.

| primers name | sequence |
| --- | --- |
| TgSQS-OE | F: TCTAGAATGGTGAGCATTGGGGCGTTAT |
|  | R: GGTACCTTACAGTTTGAAGGTGAGTATAGC |
| pET-32a-TgSQS | F: GGATCCATGGTGAGCATTGGGGC |
|  | R: GAGCTCTTACAGTTTGAAGGTGAGTATAGCG |
| pAbAi-W1 | F: GGTACCGATTAAGAAATAGAAAATGCTTCCT |
|  | R: GTCGACCTGTGTGTGATTAACTGCTTAA |
| pAbAi-W2 | F: GGTACCATCCATCATCAGGATGAACA |
|  | R: GTCGACGTCGATTTATCACAATTACACACA |
| pAbAi-W3 | F: GGTACCCAAGTTTGCTCTATTTGTAATTAT  R: GTCGACTTGCTAATCTTTAGAGAAATTAA |
| pGADT7-TgWRKY3 | F: GAATTCATGGCAAGTGGGTTAGATTTG |
|  | R: GGATCCCTATGGGTGAACTACCAGATTTG |
| pGADT7-TgWRKY6 | F: GAATTCATGGCCGGGAGTGAAGAGA |
|  | R: GGATCCTCAGGGTCCCATTTGTAGTCTC |
| pGADT7-TgWRKY7 | F: GAATTCATGGAAGGTAAGGAGTTTGAGA |
|  | R: GGATCCTCATGGCTTGTTCTCTGTGA |
| pGreenII 0800-LUC-proTgSQS | F: GTCGACACTGCGGATCGGTTTTTAT |
|  | R: AAGCTTAACCTGATTAAGCACAATTAA |
| pCAMBIA 1300-GFP-WRKY1 | F: GGATCCATGGTTGTTCAATTTTTGTGC |
|  | R: ATGTCGACTGTAAGAGCGAAACCCCTG |
| pCAMBIA 1300-GFP-WRKY2 | F: GGATCCATGGATTTCTGCGGATCG |
|  | R: ATGTCGACTAACAGATCATATAAACTGAATAAAATC |
| pCAMBIA 1300-GFP-WRKY3 | F: GGATCCATGGCAAGTGGGTTAGATTTG |
|  | R: ATGTCGACTGGGTGAACTACCAGATTTG |
| pCAMBIA 1300-GFP-WRKY4 | F: GGATCCATGGTGTCGAAGCCAACG |
|  | R: ATGTCGACTAGCAATTGCTGACCA |
| pCAMBIA 1300-GFP-WRKY6 | F: GGATCCATGGCCGGGAGTGAAGAG |
|  | R: ATGTCGACGGGTCCCATTTGTAGTCTCC |
| pCAMBIA 1300-GFP-WRKY7 | F: GGATCCATGATGGAAGGTAAGGAGTTTG |
|  | R: ATGTCGACTGGCTTGTTCTCTGTGAAG |
| pCAMBIA 1300-GFP-WRKY8 | F: GGATCCATGTATTGCAGGAGTTACTACC |
|  | R: ATGTCGACGTTTTTGCGCATTCCAG |
| pCAMBIA 1300-GFP-WRKY9 | F: GGATCCATGGCGAACAGGGGTTACTAC |
|  | R: ATGTCGACCGAATTGGGTATTTCAGTTG |
| pCAMBIA 1300-GFP-WRKY11 | F: GGATCCATGGATCTGGCTCCAGCC |
|  | R: ATGTCGACTGAAAATAAGACCAAAACCTG |
| pCAMBIA 1300-GFP-WRKY12 | F: GGATCCATGCTACAACAAGAGCTGAATC |
|  | R: ATGTCGACAGACGAGAGAATGTGGG |
| pCAMBIA 1300-GFP-WRKY13 | F: GGATCCATGGAGTACGACAACAATGG |
|  | R: ATGTCGACAGGGCCCATAAGCAATC |
| pCAMBIA 1300-GFP-WRKY14 | F: GGATCCATGGATGATCATCAGCAGC |
|  | R: ATGTCGACATGCAAAGAACCTTCATAG |
| pCAMBIA 1300-GFP-WRKY15 | F: GGATCCATGCCTCGGATTTTGTCATC |
|  | R: ATGTCGACAATTCTTCTATTTGCTTCGG |
| pCAMBIA 1300-GFP-WRKY16 | F: GGATCCATGGAGCAACGCAAAAAT |
|  | R: ATGTCGACTTGTATTGAGTTACACAAGAATT |
| pCAMBIA 1300-GFP-WRKY17 | F: GGATCCATGGAGGAGCAAGGGATG |
|  | R: ATGTCGACAGTATGCATCACAAGATTTGG |
| pCAMBIA 1300-GFP-WRKY18 | F: GGATCCATGGCCAAGGGCAATCC |
|  | R: ATGTCGACTTGGCGGTGTTCATTTCTG |
| pCAMBIA 1300-GFP-WRKY19 | F: GGATCCATGGGTGGACTTACGGATATG |
|  | R: ATGTCGACTTGGCGGTGTTCATTTCTG |
| pCAMBIA 1300-GFP-WRKY20 | F: GGATCCATGGAAGGGGACCGTATTAATG |
|  | R: ATGTCGACTTGTATGGCGTTACAAGGG |
| pCAMBIA 1300-GFP-WRKY21 | F: GGATCCATGACCAGAAACAACATGTG |
|  | R: ATGTCGACTTGTATTGAGTTACACAAGAATT |
| pCAMBIA 1300-GFP-WRKY23 | F: GGATCCATGGCAGTGGAGATGTTG |
|  | R: ATGTCGACTGGATCCACAACGAGG |
| pCAMBIA 1300-GFP-WRKY24 | F: GGATCCATGGAGGAAACAAACAAATTAC |
|  | R: ATGTCGACGATAACTGCATTGTTCAAGC |
| pCAMBIA 1300-GFP-WRKY26 | F: GGATCCATGGAGTTAATTTGCAGAGACAC |
|  | R: ATGTCGACACCGCCTTGGTTATAACC |
| pCAMBIA 1300-GFP-WRKY29 | F: GGATCCATGGCTGCCAGTGTGGAGAAAATG |
|  | R: ATGTCGACCTAACATTTCCTCGTCTCTTTCCTG |

Table S2. Primers of sequences for qRT-PCR analysis.

| gene | primer |
| --- | --- |
| TgSQS | F: GGTGAGCATTGGGGCGTTAT |
|  | R: AAGTCCGGATCTGACGGGAT |
| TgWRKY3 | F: TCACTGCTCAAAACGGAGGA |
|  | R: CTGGCAGGACAACCTCTCAT |
| TgWRKY6 | F: AGCAGTCTCCAACAACAGGG |
|  | R: TAGAGGGGATAGGCACGAGG |
| TgWRKY7 | F: AATTGTGTTGTAGCGGGGGA |
|  | R: TTTGCAAGGGATGAGAGGGG |

Table S3. Summary of transcriptome sequencing for *Torreya grandis*.

| Sample | Raw Reads | Clean Reads | Clean Bases | Error (%) | Q20(%) | Q30(%) | GC Content (%) |
| --- | --- | --- | --- | --- | --- | --- | --- |
| CK-1 | 21128225 | 20617797 | 6.19G | 0.02 | 98.14 | 94.09 | 43.78 |
| CK-2 | 24136215 | 23218348 | 6.97G | 0.02 | 98.13 | 94.15 | 44.59 |
| CK-3 | 23133432 | 22377682 | 6.71G | 0.03 | 98.1 | 94.06 | 44.54 |
| D40-1 | 23627025 | 22650588 | 6.80G | 0.02 | 98.17 | 94.3 | 45.09 |
| D40-2 | 22661550 | 21771586 | 6.53G | 0.03 | 98 | 93.99 | 44.62 |
| D40-3 | 24005662 | 23260241 | 6.98G | 0.02 | 98.14 | 94.15 | 44.36 |
| D60-1 | 21971362 | 21168429 | 6.35G | 0.02 | 98.75 | 95.98 | 44.95 |
| D60-2 | 23735807 | 22979890 | 6.89G | 0.02 | 98.78 | 95.99 | 44.49 |
| D60-3 | 22994741 | 22288759 | 6.69G | 0.02 | 98.73 | 95.84 | 44.79 |

Table S4. Statistics of *Torreya grandis* splicing transcriptome.

|  | Total | Min length | Mean  length | Median length | Max length | N50 | N90 |
| --- | --- | --- | --- | --- | --- | --- | --- |
| Transcripts | 158990 | 301 | 1563 | 989 | 18018 | 2532 | 648 |
| Genes | 66798 | 301 | 1241 | 668 | 18018 | 2154 | 479 |

Table S5. Functional annotation of the *Torreya grandis* transcriptome.

| Database | Number of Unigenes | percentage |
| --- | --- | --- |
| KOG | 6291 | 9.41 |
| KO | 9405 | 14.07 |
| NT | 15385 | 23.03 |
| SwissProt | 21671 | 32.44 |
| GO | 23627 | 35.37 |
| PFAM | 23631 | 35.37 |
| NR | 27983 | 41.89 |
| Annotated in at least one Database | 33318 | 49.87 |
| Total Unigenes | 66798 | 100 |

Table S6. FPKM value of sterol biosynthesis pathway related genes in the transcriptome of *Torreya grandis* seedlings.

| ID | Name | CK-1 | CK-2 | CK-3 | D40-1 | D40-2 | D40-3 | D60-1 | D60-2 | D60-3 |
| --- | --- | --- | --- | --- | --- | --- | --- | --- | --- | --- |
| Cluster-1843.14996 | DXS | 27.59 | 42.46 | 32.31 | 61.68 | 39.54 | 45.29 | 152.4 | 226.35 | 165.15 |
| Cluster-1843.17433 | DXR | 29.25 | 34.76 | 31.33 | 38.61 | 29.07 | 30.73 | 16.49 | 16.37 | 11.95 |
| Cluster-1843.12041 | HMGS | 2.06 | 2 | 2.66 | 1.79 | 1.74 | 1.3 | 71.91 | 88.81 | 109.11 |
| Cluster-1843.20890 | HMGS | 14.67 | 12.41 | 11.42 | 12.41 | 12.7 | 12.46 | 154.83 | 205.33 | 244.4 |
| Cluster-1843.12154 | HMGR | 0 | 0 | 0 | 0.8 | 0.32 | 0 | 177.45 | 381.18 | 270.28 |
| Cluster-1843.14430 | HMGR | 11.29 | 13.6 | 14.66 | 26.39 | 18.78 | 21.57 | 502.14 | 841.67 | 792.34 |
| Cluster-1843.14431 | HMGR | 9.46 | 8.63 | 14.33 | 27.23 | 20.08 | 18.76 | 3510.09 | 5249.25 | 3715.34 |
| Cluster-1843.16325 | HMGR | 19.69 | 16.93 | 21.97 | 38.1 | 26.43 | 24.28 | 1811.62 | 2485.97 | 1938.56 |
| Cluster-1843.20098 | HMGR | 11.86 | 8.93 | 18.45 | 24.84 | 21.45 | 21.73 | 3630.65 | 5130.27 | 3698.79 |
| Cluster-1843.20100 | HMGR | 36.83 | 31.78 | 39.25 | 64 | 58.5 | 49.13 | 1951.91 | 2922.56 | 2497.24 |
| Cluster-1843.24403 | HMGR | 6.1 | 3.05 | 5.55 | 8.89 | 6.66 | 4.96 | 2984.8 | 4354.12 | 3078.08 |
| Cluster-1843.28970 | HMGR | 3.86 | 4.75 | 2.86 | 0 | 8.55 | 1.08 | 96.62 | 174.98 | 120.86 |
| Cluster-1843.16766 | MK | 2.47 | 1.84 | 2.67 | 3.2 | 2.86 | 2.43 | 15.03 | 18.66 | 18.48 |
| Cluster-1843.18487 | CAS | 21.01 | 20.18 | 23.44 | 15.08 | 14.75 | 16.02 | 14.94 | 16.46 | 13.88 |
| Cluster-1843.27216 | DWF1 | 12.64 | 12.81 | 14.41 | 12.46 | 14.79 | 15.05 | 12.5 | 13.75 | 17.64 |
| Cluster-1843.5911 | DWF1 | 2.77 | 0.57 | 3.04 | 2.3 | 6.63 | 0.62 | 5.12 | 8.53 | 5.27 |
| Cluster-1843.15850 | SQS | 9.14 | 9.25 | 8.52 | 8.89 | 11.86 | 12.6 | 11.48 | 10.96 | 13.33 |
| Cluster-1843.22038 | FPPS | 5.19 | 2.67 | 4.41 | 3.35 | 3.07 | 2.86 | 7.81 | 10.41 | 11.41 |
| Cluster-1843.20742 | IPPI | 32.38 | 25.83 | 27.71 | 27.53 | 29.45 | 25.91 | 42.93 | 47.56 | 52.5 |
| Cluster-1843.15165 | SQE | 8.31 | 8.91 | 7.71 | 4.95 | 6.59 | 6.44 | 2.69 | 2.31 | 2.55 |
| Cluster-1843.18154 | SQE | 25.06 | 34.24 | 24.34 | 12.58 | 16.11 | 15.98 | 5.12 | 4.83 | 5.68 |
| Cluster-1843.18726 | SQE | 99.42 | 107.46 | 116.85 | 103.94 | 93.03 | 126.01 | 96.24 | 105.56 | 84.69 |
| Cluster-1843.19828 | SQE | 35.93 | 27.04 | 31.02 | 30.42 | 28.83 | 34.36 | 14.32 | 16.5 | 14.48 |
| Cluster-1843.20782 | SQE | 31.59 | 31.96 | 28.51 | 34.62 | 39.27 | 38.07 | 4.9 | 5.86 | 6.84 |
| Cluster-1843.23279 | SQE | 3.15 | 1.39 | 5.93 | 5.43 | 4.94 | 3.92 | 145.44 | 144.63 | 113.88 |
| Cluster-1843.23984 | SQE | 11.2 | 16.13 | 14.28 | 12.92 | 12.73 | 11.94 | 13.49 | 15.99 | 15 |
| Cluster-1843.29350 | SQE | 2.11 | 3.25 | 1.74 | 2.03 | 0.8 | 1.69 | 11.86 | 30.21 | 28.45 |
| Cluster-1843.20243 | SMT1 | 24.25 | 23.28 | 24.25 | 32.04 | 39.74 | 36.1 | 28.54 | 28.23 | 26.09 |
| Cluster-7018.0 | SMT1 | 0 | 0.66 | 1.15 | 0.69 | 0.46 | 0.22 | 0 | 0 | 0 |
| Cluster-1843.21008 | SMT1 | 23.18 | 30.84 | 27.14 | 23.89 | 22.73 | 23.05 | 27.44 | 33.57 | 32.56 |
| Cluster-1843.21419 | SMO2 | 9.97 | 8.56 | 8.99 | 9.51 | 11.58 | 8.31 | 4.12 | 2.88 | 3.03 |
| Cluster-1141.0 | SMO2 | 0 | 0 | 0 | 0 | 0 | 0 | 3.32 | 0 | 0.88 |
| Cluster-1843.27291 | SMO1 | 13.71 | 12.57 | 10.53 | 6.06 | 8.25 | 7.37 | 3.49 | 4.4 | 5.22 |
| Cluster-1843.11602 | CEC1 | 14.36 | 12.58 | 12.23 | 13.33 | 11 | 9.44 | 9.37 | 10.16 | 8.69 |
| Cluster-1843.30829 | CEC1 | 1.22 | 1.29 | 1.18 | 1.12 | 1.15 | 1.07 | 0.48 | 0.39 | 0.56 |
| Cluster-1843.5942 | FK | 5.51 | 5.03 | 5.53 | 4.74 | 11.89 | 3.42 | 3.63 | 3.88 | 4.82 |
| Cluster-1843.23140 | STE1 | 2.44 | 0.85 | 1.52 | 0.13 | 0.43 | 0.81 | 0.35 | 0.04 | 0.51 |

CAS, cycloartenol synthase; CEC1, cycloeucalenol cycloisomerase; DWF1, D24 sterol reductase; DXR, 1-deoxy-D-xylulose-5-phosphate reductoisomerase; DXS, 1-deoxy-D-xylulose-5-phosphate synthase; FK, Δ^14^-sterol reductase; FPPS, FPP synthase; HMGR, 3-hydroxy-3-methylglutaryl-CoA reductase; HMGS, 3-hydroxy-3-methylglutaryl-CoA synthase; IPPI, isopentenyl diphosphate isomerase; MK, mevalonate kinase; SMO1, sterol-4a-methyl oxidase 1; SMO2, sterol-4a-methyl oxidase 2; SMT1, sterol methyltransferase 1; SQE, squalene monooxygenase/epoxidase; SQS, squalene synthase; STE1, C-5 sterol desaturase.


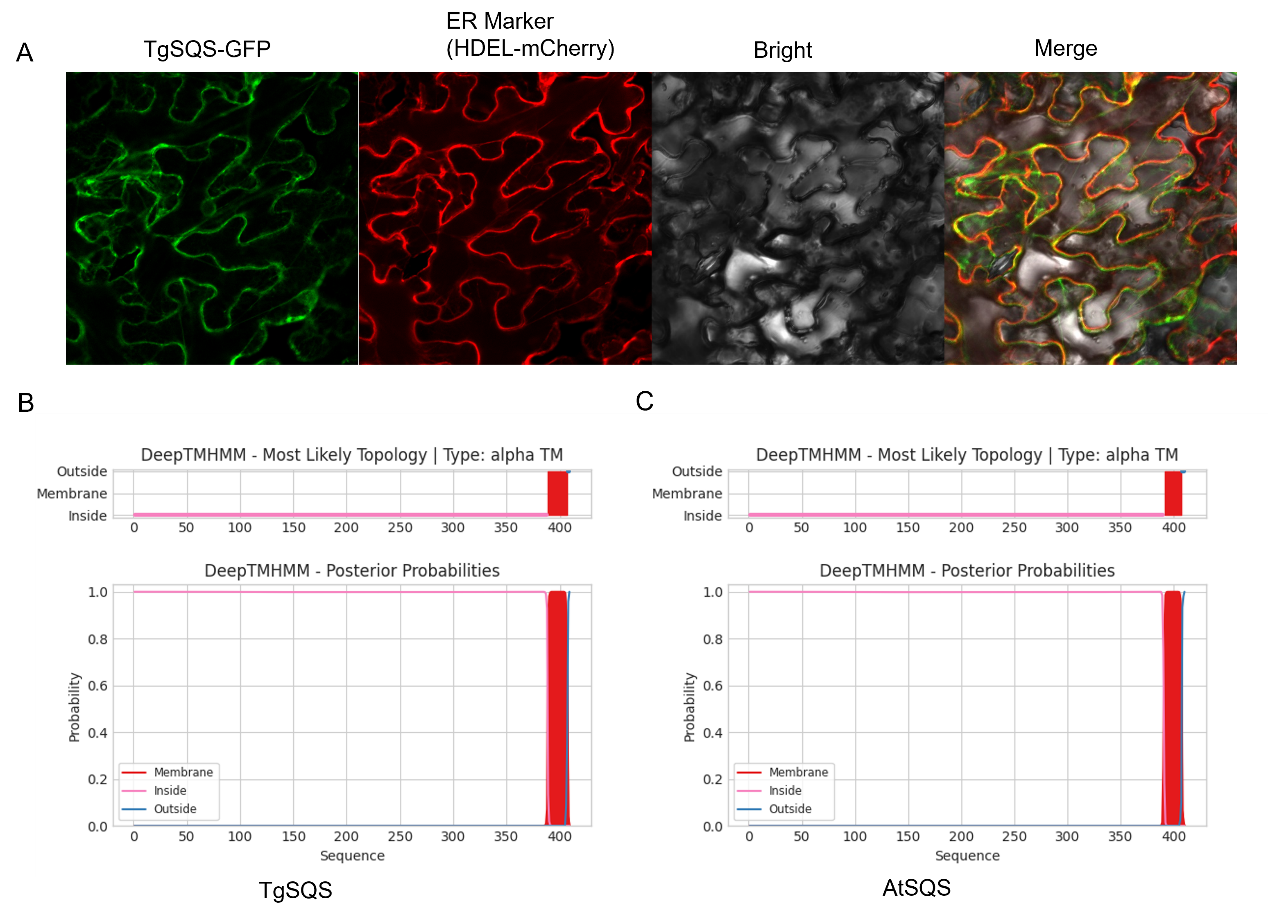


Figure S1. Subcellular localization and transmembrane domain prediction of TgSQS. (A) Confocal images showing the subcellular localization of TgSQS-GFP in *Nicotiana benthamiana* leaves. HDEL-mCherry was used as an ER marker. ER, endoplasmic reticulum. Scale bar = 50 μm. (B-C) Predicted transmembrane domains of *Torreya grandis* and *Arabidopsis thaliana* SQS.


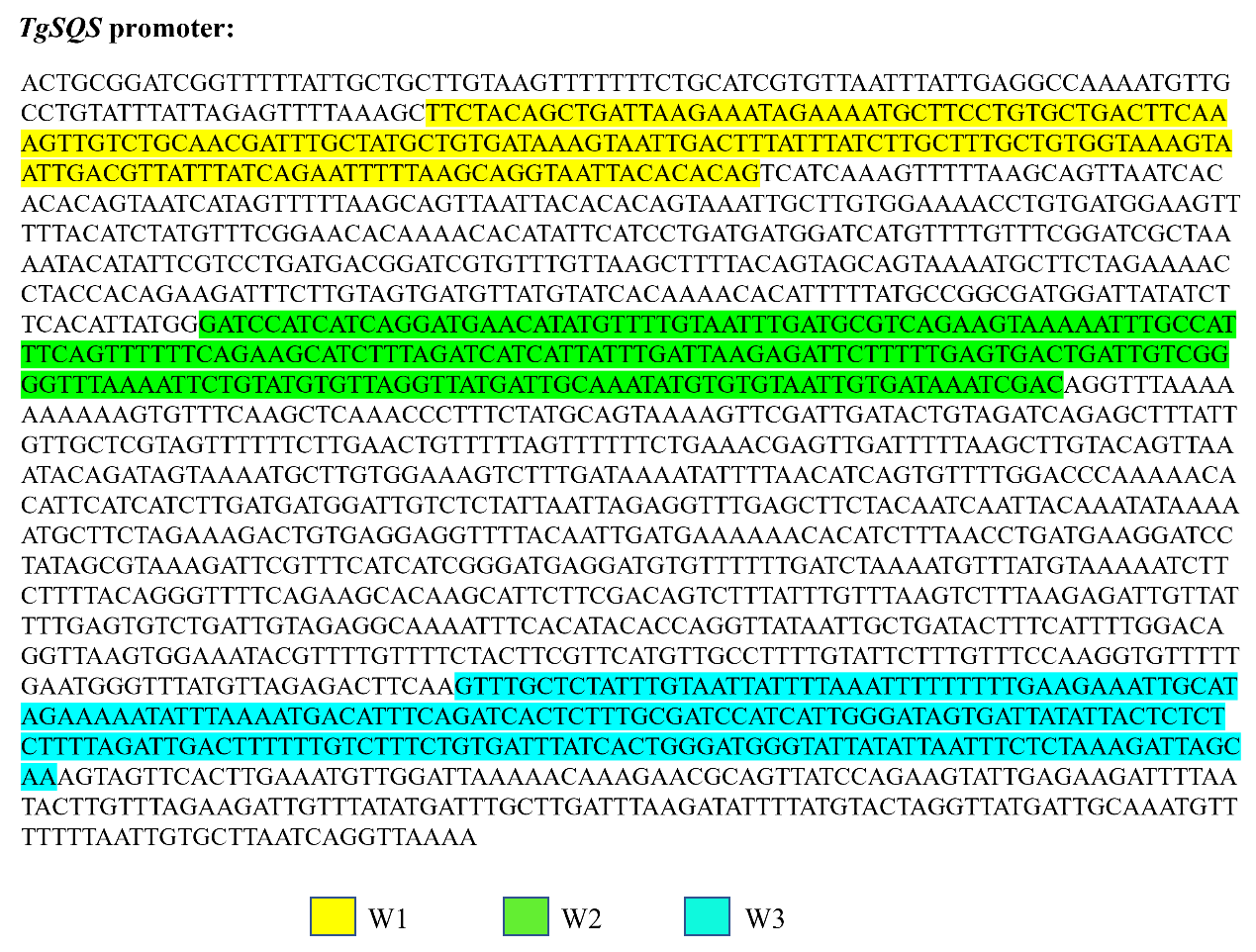


Figure S2. The nucleotide sequence of TgSQS promoter (1874 bp before ATG, excluding ATG). The nucleotide sequences of W1, W2, and W3 (200 bp sequence containing W box) are labeled with yellow, green, and cyan colors, respectively.


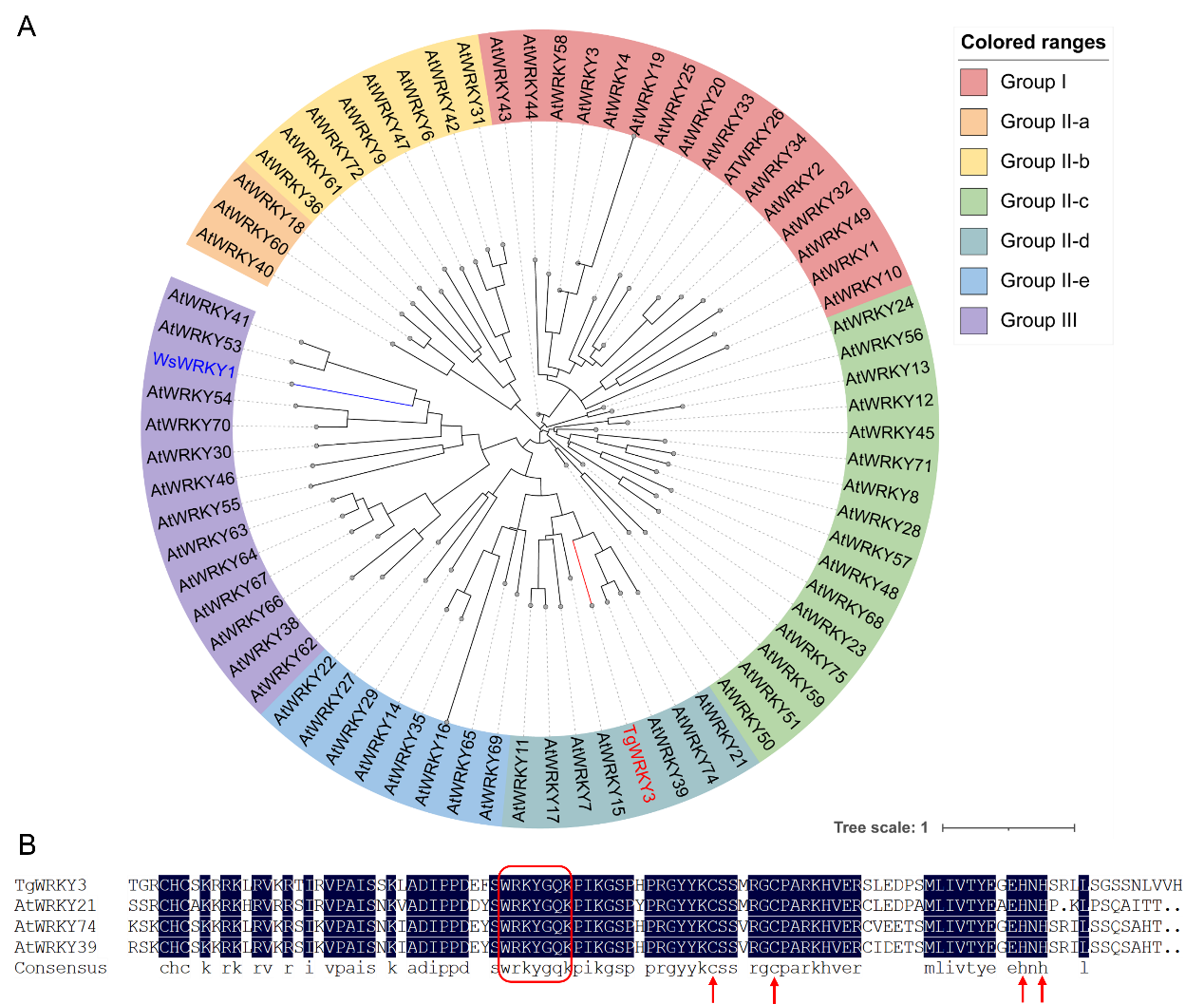


Figure S3. The analysis of TgWRKY3 amino acid sequence. (A) Construction of phylogenetic tree of TgWRKY3 and WsWRKY1 from AtWRKYs using neighbor-joining method; (B) Alignment of the full-length amino acid sequences of TgWRKY3 and its homologs in *Arabidopsis thaliana*. The red box is WRKY conservative domain, and the red arrow represents the characteristic zinc finger domain.


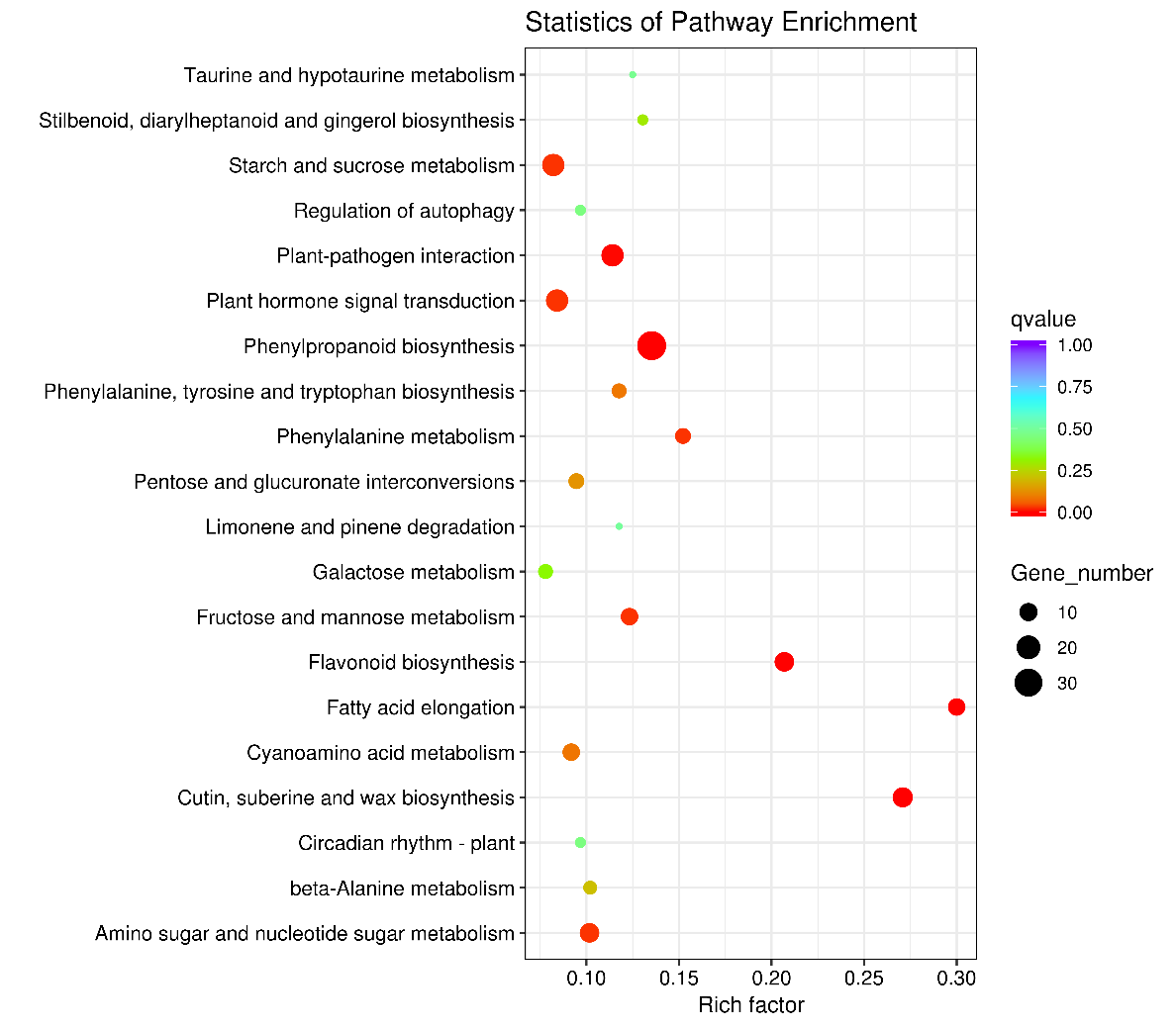


Figure S4. KEGG path enrichment scatter plot D40 vs CK. The vertical axis represents the name of the path, and the horizontal axis represents the rich factor corresponding to the path. The size of qvalue is represented by the color of the dot. The smaller the qvalue is, the closer the color is to red. The number of differential genes contained in each path is represented by the size of the dot.


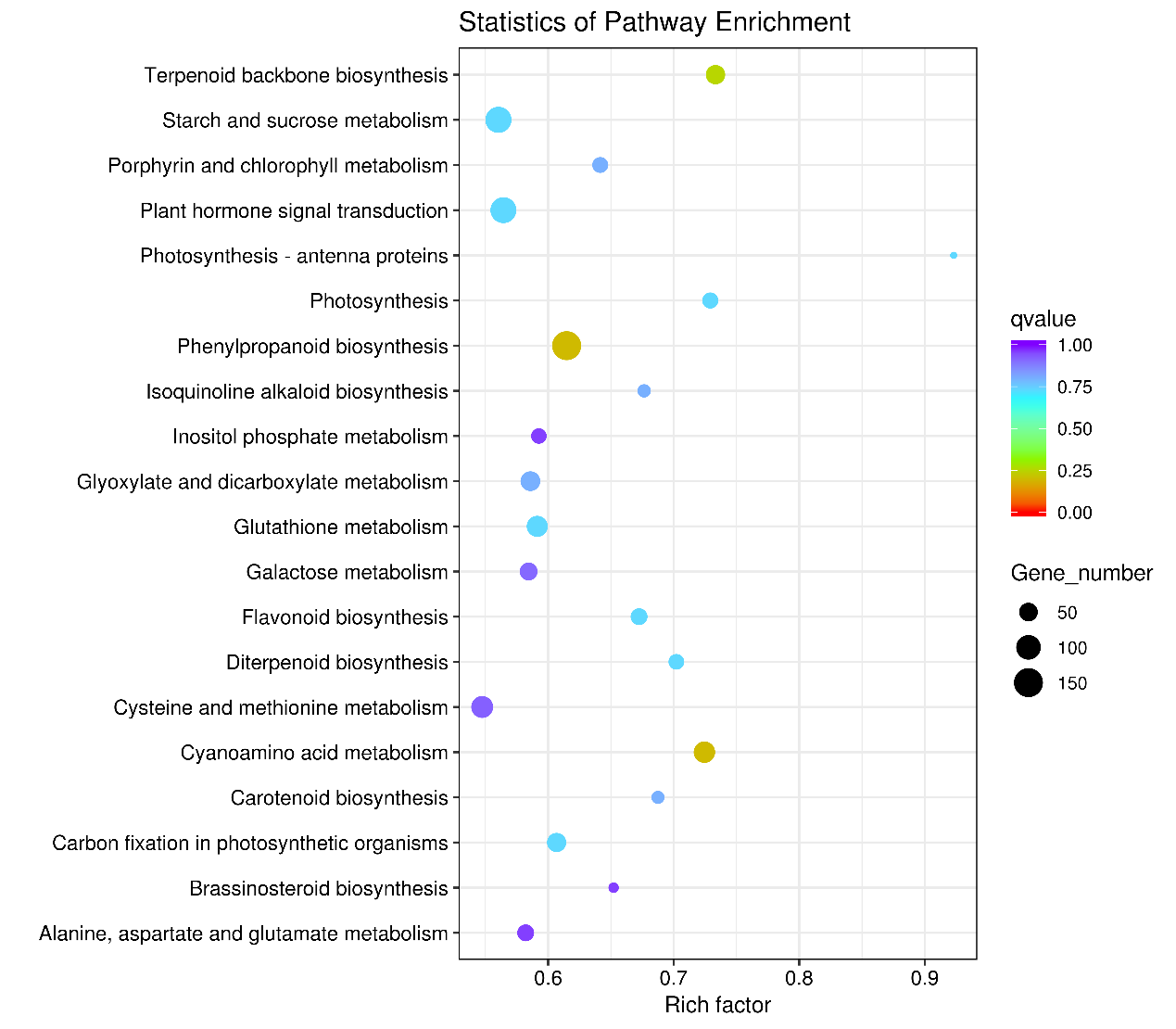
Figure S5. KEGG path enrichment scatter plot D60 vs CK. The vertical axis represents the name of the path, and the horizontal axis represents the rich factor corresponding to the path. The size of qvalue is represented by the color of the dot. The smaller the qvalue is, the closer the color is to red. The number of differential genes contained in each path is represented by the size of the dot.


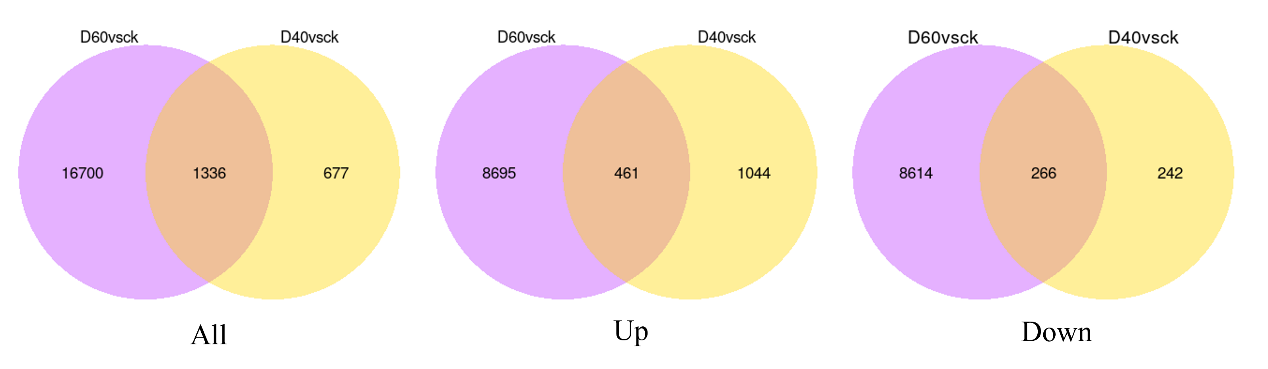


Figure S6. Venn diagrams showed the numbers of the DEGs in three comparisons.
